# The Macrophage Reprogramming Ability of Antifolates Reveals Soluble CD14 as a Potential Biomarker for Methotrexate Response in Rheumatoid Arthritis

**DOI:** 10.3389/fimmu.2021.776879

**Published:** 2021-11-05

**Authors:** Sara Fuentelsaz-Romero, Celia Barrio-Alonso, Raquel García Campos, Mónica Torres Torresano, Ittai B. Muller, Ana Triguero-Martínez, Laura Nuño, Alejandro Villalba, Rosario García-Vicuña, Gerrit Jansen, María-Eugenia Miranda-Carús, Isidoro González-Álvaro, Amaya Puig-Kröger

**Affiliations:** ^1^Unidad de Inmunometabolismo e Inflamación, Instituto de Investigación Sanitaria Gregorio Marañón, Hospital General Universitario Gregorio Marañón, Madrid, Spain; ^2^Amsterdam Rheumatology and Immunology Center, Amsterdam University Medical Center, Vrije Universiteit, Amsterdam, Netherlands; ^3^Servicio de Reumatología, Hospital Universitario La Princesa, Instituto de Investigación Sanitaria Hospital Universitario La Princesa, Madrid, Spain; ^4^Department of Rheumatology, Hospital Universitario La Paz-IdiPaz, Madrid, Spain

**Keywords:** macrophages, methotrexate, pemetrexed, sCD14, predictor biomarker

## Abstract

The identification of “trained immunity/tolerance” in myeloid cells has changed our perception of the performance of monocytes and macrophages during inflammatory and immune responses. Pemetrexed (PMX) and methotrexate (MTX) are blockers of the one-carbon metabolism (OCM) and commonly used therapeutic agents in cancer and rheumatoid arthritis (RA). We have previously showed that MTX promotes trained immunity in human macrophages. In the present manuscript, we have assessed the anti-inflammatory effects of PMX and MTX and found that OCM blockers alter the functional and gene expression profile of human macrophages and that OCM blockade reprograms macrophages towards a state of lipopolysaccharide (LPS) tolerance at the signaling and functional levels. Moreover, OCM blockade reduced macrophage LPS responsiveness by impairing the expression of membrane-bound and soluble CD14 (sCD14). The therapeutic relevance of these results was later confirmed in early RA patients, as MTX-responder RA patients exhibit lower sCD14 serum levels, with baseline sCD14 levels predicting MTX response. As a whole, our results demonstrate that OCM is a metabolic circuit that critically mediates the acquisition of innate immune tolerance and positions sCD14 as a valuable tool to predict MTX response in RA patients.

## Introduction

Folates are one-carbon donors in biosynthetic pathways like *de novo* synthesis of purines and thymidylate, amino acid metabolism, and DNA methylation (1, 2). One-carbon metabolism (OCM) dispenses carbon atoms between various acceptor molecules required for biosynthesis and contributes to energy balance by providing ATP and NADPH (3). Accordingly, antifolates [including methotrexate (MTX) and pemetrexed (PMX)] are potent inhibitors of folate-dependent enzymes engaged in nucleotide synthesis and inhibit DNA replication (2). PMX is a therapeutically relevant antifolate used in combination with cisplatin or carboplatin as the standard first-line treatment of malignant pleural mesothelioma (4, 5), advanced non-squamous non-small cell lung cancer (NSCLC) (6) and in maintenance therapy for NSCLC. PMX is transported into the cells *via* the reduced folate carrier (RFC; *SLC19A1*) and, unlike MTX, is an excellent substrate for the proton-coupled folate transporter (PCFT; *SLC46A1*) (7). PMX is also an efficient substrate for the enzyme folylpoly-γ glutamate synthetase (FPGS) that catalyzes the addition of multiple glutamyl residues to the γ-carboxyl on the terminal glutamate of both folates and antifolates upon entry into the cell (8). PMX polyglutamylation enhances both its intracellular retention and its inhibitory potency towards thymidylate synthase (TS) and glycinamide ribonucleotide formyltransferase (GARFT) (8).

Rheumatoid arthritis (RA) is an autoimmune systemic disease affecting mainly diarthrodial joints, commonly treated with weekly administered MTX as the main starting therapy and anchor drug (9, 10). The current paradigm in the treatment of RA is to start treatment with disease-modifying anti-rheumatic drugs (DMARDs) as soon as possible, in order to take advantage of the “window of opportunity,” a period at the beginning of the disease in which the response to effective treatment is disproportionately greater, increasing the possibility to achieve remission (11). On the contrary, PMX is not approved for RA treatment, in spite of the fact that it reduces inflammatory cell infiltration in joints, limits cartilage-bone destruction, and lowers serum TNFα and IL-17 in a rat model of collagen-induced arthritis (12).

Macrophages exhibit innate immune memory whereby they are durably primed by certain stimuli for enhanced or diminished responses to secondary stimuli (training/tolerance) (13–16). Macrophages are abundant in the synovium of RA joints, where they contribute to pathogenesis through the production of proinflammatory mediators (17–19). We have previously shown that macrophages from human RA joints exhibit a transcriptomic and phenotypic proinflammatory polarization profile that resembles that of granulocyte-macrophage colony-stimulating factor (GM-CSF)-differentiated macrophages (GM-MØ) (20) and that MTX conditions macrophages towards the acquisition of a state of tolerance that renders them less responsive to TLR ligands, TNFα, and RA synovial fluid (21, 22).

Low-dose MTX is safe, inexpensive, and effective, but 30%–40% of RA patients discontinue MTX treatment due to intolerance, inefficacy, or loss of clinical responsiveness at later time points (MTX resistance) (23, 24). Considering the “window of opportunity” concept, it would be interesting to have biomarkers predicting MTX response, therefore avoiding a delay in achieving remission in those MTX-non-responder patients. On the other hand, given these therapeutic limitations of MTX, and considering the widespread use of PMX in hyper-proliferative pathologies, we have now explored the phenotypic and functional effects of PMX on human macrophages. Our results indicate that PMX impairs TLR4 signaling and cytokine production in macrophages and that these effects are dependent on the loss of membrane and soluble CD14 (sCD14) expression. The pathological significance of these findings has been confirmed in MTX-treated RA patients, which exhibit significantly reduced sCD14 levels. Altogether, our results indicate that PMX functionally reprograms human macrophages, opening further research of this drug to new opportunities beyond the limit of its actual clinical utility, and demonstrate that innate tolerance can be induced in human macrophages through blockade of the OCM.

## Materials and Methods

### Cell Culture

Human peripheral blood mononuclear cells (PBMCs) were isolated from buffy coats from normal donors over a Lymphoprep (Nycomed Pharma) gradient. Monocytes were purified from PBMCs by magnetic-activated cell sorting using CD14 microbeads (Miltenyi Biotech). Monocytes were cultured at 0.5 × 10^6^ cells/ml for 7 days in Roswell Park Memorial Institute (RPMI) 1640 (standard RPMI, which contains 1 mg/L folic acid) supplemented with 10% fetal calf serum, at 37°C in a humidified atmosphere with 5% CO_2_, and containing GM-CSF (1,000 U/ml) to generate GM-CSF-polarized macrophages (GM-MØ) or M-CSF (10 ng/ml) to generate M-CSF-polarized macrophages (M-MØ). GM-CSF or M-CSF was added every 2 days. Low-dose PMX (50 nM), MTX (50 nM), and/or folinic acid (FA; 5-formyl tetrahydrofolate, 5 µM), pifithrin-α cyclic (25–50 μM) was added once on monocytes together with GM-CSF or M-CSF. When indicated, lipopolysaccharide (LPS) (10 ng/ml, 0111:B4 strain, smooth LPS (sLPS) that exclusively binds TLR4, InvivoGen) or LTA (5 µg/ml, from *Staphylococcus aureus*, InvivoGen) was added at the indicated time points onto 7-day fully differentiated macrophages without replacing the culture medium. sCD14 (20–200 ng/ml, BioLegend) was added on macrophages for intracellular signaling measurements (15 min) and IL-6 detection (3 h).

### Rheumatoid Arthritis Patients

#### Discovery Cohort

Peripheral blood was obtained from 10 early RA patients who fulfilled the 2010 American College of Rheumatology (ACR) revised criteria (25), with a disease duration of less than 6 months and who had never received disease-modifying drugs or corticosteroids. Disease activity was assessed using the DAS28 score (26). Plasma concentrations of sCD14 were determined by ELISA at the basal visit and 6 months after initiating treatment with weekly low-dose methotrexate (15–25 mg/week); none of the patients were taking prednisone at the time of the first or second determination. All of the included patients had a good clinical response to MTX (nine had achieved remission as determined by a DAS28 <2.6, and the remaining one patient had persistent low activity with a DAS28 >1.2) and, accordingly, had discontinued prednisone at least 1 month prior to the 6-month sCD14 determination. Patients who required continued treatment with prednisone were not included in order to eliminate the possible interference of this immunomodulatory drug with the results. In sCD14 ELISA, interference of rheumatoid factor was ruled out after confirming that serial dilutions of plasma yielded identical sCD14 levels after adjusting for the diluting factor (27). The study was approved by the Hospital La Paz-IdiPAZ Ethics Committee, and all subjects provided written informed consent according to the Declaration of Helsinki.

#### Validation Cohort

For sCD14 determination in serum, early arthritis patients in MTX monotherapy were recruited from the Princesa Early Arthritis Register Longitudinal (PEARL) study. The PEARL study comprises patients with one or more swollen joint and symptoms with ≤1 year of evolution. The register protocol includes four visits during a 2-year follow-up (0, 6, 12, and 24 months). Sociodemographic, clinical, therapeutic, and laboratory data are recorded and included in an electronic database. Biological samples are collected at each visit and stored at −80°C in the Instituto de Investigación Sanitaria La Princesa (IIS-IP) Biobank for translational research. More detailed description of PEARL protocol has been previously published (28). None of the selected patients received corticosteroids prior to baseline visit nor during the first 6 months of follow-up. Response was considered if at the 6-month visit disease activity level decreased to remission or low disease activity provided that swollen joint count must have decreased, as well as three out of the four following variables must have decreased: tender joint count, erythrocyte sedimentation rate, C-reactive protein (CRP), and global disease activity by patient. Non-responders were considered if the disease activity level worsened from baseline to the 6-month visit. The study was approved by the Hospital La Princesa Ethics Committee (PI-518, March 28, 2011), and all subjects provided written informed consent according to the Declaration of Helsinki. Clinical and demographical data for discovery (Hospital La Paz) and validation (Hospital La Princesa) cohorts are as follows:

**Table:** 

Hospital La Paz	Responder
	(n = 10)
Female; n (%)	6 (60)
Age; p50 [p25–p75]	60.5 (44.25–71.75)
RF positive; n (%)	6 (60)
ACPA positive; n (%)	1 (10)
DAS28; p50 [p25–p75]	4.97 (3.79–5.56)
HAQ; p50 [p25–p75]	1.02 (0.67–1.67)
Dose methotrexate (mg); p50 [p25–p75]	17.5 (15–20)

**Table:** 

Hospital La Princesa	Responder	Non-responder	*p*-Value
	(n = 23)	(n = 10)	
Female; n (%)	20 (86.96)	9 (90)	1
Age; p50 [p25–p75]	56.57 (42.69–67.48)	59.66 (53.62–76.84)	0.22
RF positive; n (%)	14 (60.87)	7 (70)	0.46
ACPA positive; n (%)	11 (47.83)	6 (60)	0.39
DAS28; p50 [p25–p75]	4.90 (4.07–5.81)	4.11 (3.46–5.09)	0.12
HAQ; p50 [p25–p75]	1.37 (0.87–1.75)	0.87 (0.5–1.75)	0.32
Dose methotrexate (mg); p50 [p25–p75]	15 (15–20)	15 (12.5–15)	0.27

p50, median; p25-p75, interquartile range; RF, rheumatoid factor; ACPA, anti-citrullinated protein antibodies; DAS28, disease activity score estimated with 28 joint count; HAQ, health assessment questionnaire.

### RNAseq and Gene Set Enrichment Analysis

Total RNA was isolated from three independent preparations and processed at BGI (https://www.bgitechsolutions.com), where library preparation, fragmentation, and sequencing were performed using the BGISEQ-500 platform. An average of 5.41-Gb bases were generated per sample; and after filtering, clean reads were mapped to the reference (UCSC Genome assembly hg38) using Bowtie2 (average mapping ratio 93.41%) (29). Gene expression levels were calculated by using the RSEM software package (30). Differential gene expression was assessed by using DEseq2 algorithms using the parameters Fold change >2 and adjusted *p*-value <0.05. For gene set enrichment analysis (GSEA) (http://software.broadinstitute.org.gsea/index.jsp), the gene sets available at the website (31), as well as the “p53_Target_Gene” gene set, that contains the top 98 genes identified as p53 targets, were used (32). The data discussed in this publication have been deposited in Gene Expression Omnibus (GEO) of National Center for Biotechnology Information (NCBI) (33) and are accessible through GEO series accession numbers GSE159349 and GSE159380. For microarray data, statistical analysis for differential gene expression was carried out using empirical Bayes moderated t-test implemented in the limma package (http://www.bioconductor.org). The transcriptome of MTX-GM-MØ has been previously described (21) and is available at GEO (GSE71253).

### Quantitative Real-Time RT-PCR

Total RNA was retrotranscribed, and cDNA was quantified using the Universal Human Probe Roche library (Roche Diagnostics). Quantitative real-time PCR (qRT-PCR) was performed on a LightCycler^®^ 480 (Roche Diagnostics). Assays were made in triplicates, and results normalized according to the expression levels of TBP. Results were obtained using the ΔΔCT method for quantitation. The oligonucleotides used to quantify mRNA transcripts were (5′–3′) as follows: CD14 forward gttcggaagacttatcgaccat; CD14 reverse acaaggttctggcgtggt; IL1β forward ctgtcctgcgtgttgaaaga; IL1β reverse ttgggtaatttttgggatctaca; IL6 forward gatgagtacaaaagtcctgatcca; IL6 reverse ctgcagccactggttctgt; TBP forward cggctgtttaacttcgcttc; and TBP reverse cacacgccaagaaacagtga.

### RNA Interference

Two different siRNAs for TS (*TYMS* silencer select s14538 and s14539) and one control siRNA (silencer select Negative Control #1) (Ambion, Life Technologies) were used at 100 nM. GM-MØ cells were transfected with HiPerFect transfection reagent (QIAGEN), treated with PMX, and, 48 h later, tested for CD14 mRNA levels by qRT-PCR.

### Enzyme-Linked Immunosorbent Assay

Supernatants from GM-MØ were tested for the presence of IL-6, IL-10, TNFα, and CXCL10 (BioLegend) and IFNβ (R&D Systems). For the detection of sCD14 in supernatants, serum, and plasma of RA patients and healthy controls, the Human CD14 Quantikine ELISA Kit (R&D Systems) was used.

### Western Blotting

Cell lysates were obtained in radioimmunoprecipitation assay (RIPA) buffer containing 1 mM of PIC (Protease Inhibitor Cocktail, SIGMA), 10 mM of NaF, 1 mM of Na_3_VO_4_, and 0.5 mM of DTT. Cell lysate measuring 10 μg was subjected to sodium dodecyl sulfate–polyacrylamide gel electrophoresis (SDS-PAGE) and transferred onto an Immobilon polyvinylidene difluoride membrane (Millipore). Protein detection was carried out using mouse monoclonal Ab against IκBα (clone L35A5, #4814; Cell Signaling), rabbit polyclonal against p-p38 (clone D3F9), pJNK (clone 81E11) and pERK (clone D13.14.4E, #9910; Cell Signaling), p-IRF3 (clone 4D4G, #4947; Cell Signaling), and p-STAT1 (clone D4A7, #7649; Cell Signaling). Protein loading was normalized using an antibody against GAPDH (clone 6C5, sc-32233, Santa Cruz Biotechnology) or against human Vinculin (clone VIN-11-5, #V4505; Sigma-Aldrich).

### Flow Cytometry

Phenotypic analysis was carried out by immunofluorescence using standard procedures. Mouse monoclonal antibodies used for cell-surface staining included fluorescein isothiocyanate (FITC)-labeled anti-CD14 (clone MφP9; BD) and PE-labeled anti-TLR4 (clone HTA125; BioLegend). Isotype-matched labeled antibodies were included as negative controls.

### Statistical Analysis

For the analysis of clinical data, Stata 14.0 for Windows (Stata Corp LP, College Station, TX, USA) was used. For the discovery cohort, a non-parametric Wilcoxon test was used to assess the statistical significance of differences between pre- and posttreatment RA plasma sCD14. For the validation cohort, most quantitative variables followed a non-normal distribution, so they were represented as median and interquartile range (IQR), and the Mann–Whitney or Kruskal–Wallis tests were used to analyze significant differences. Qualitative variables were described using a calculation of the proportions, and Fisher’s exact test was used to compare categorical variables. To assess the ability of either baseline CD14 (sCD14) or the variation in sCD14 between baseline and 6 months’ follow-up visits (ΔsCD14) to discriminate between MTX-responder and MTX-non-responder patients, we generated receiver operating characteristic (ROC) curves through the command roctab and the option graph. Each cutoff point was selected on the basis of the best trade-off values between sensitivity, specificity, cases correctly classified, and positive (LR+) and negative (LR−) likelihood ratios reported using the command roctab with the option detail. With the use of these cutoff values, new variables were generated to define patients with high or low baseline sCD14 and those with relevant sCD14 decrease. Then, we estimated the ORs and their 95% CI with the command cs of Stata, with the option or. This command also provides the statistical significance with Fisher’s exact test.

To estimate with a better precision the capability of sCD14 to discriminate between MTX-responder and non-responder patients, we performed a multivariable logistic regression analysis in which the dependent variable was response to MTX, and as independent variables, gender, age, and baseline DAS28 were included, which are well-known predictors of treatment response in RA. Then, we consecutively included sCD14 or ΔsCD14 in the model to determine their respective ORs adjusted by age, gender, and baseline DAS28.

Statistical analysis for the rest of the figures was done using GraphPad Prism, using parametric Student’s t-test, as appropriate, and one-way ANOVA test coupled with Tukey’s post-hoc test, where indicated. *p*-Value <0.05 was considered significant (^*^p < 0.05; ^**^p < 0.01; ^***^p < 0.001).

## Results

### Pemetrexed Induces a Proinflammatory Profile in Granulocyte-Macrophage Colony-Stimulating Factor-Primed Macrophages

**Figure 1:**
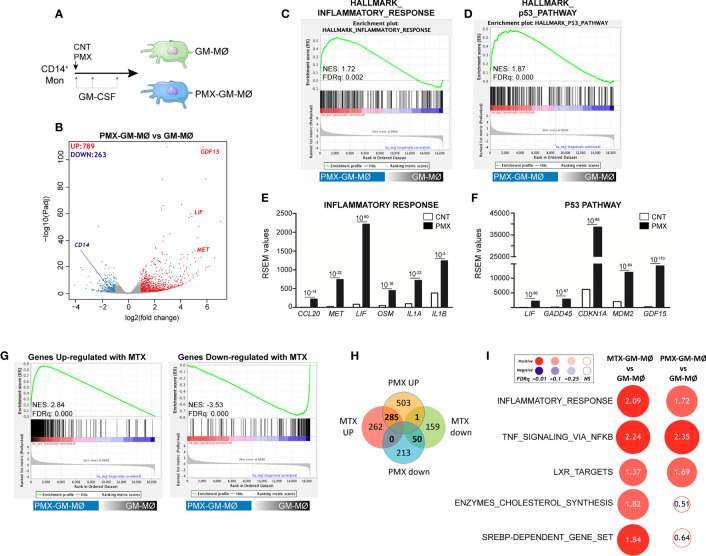
PMX promotes the acquisition of a proinflammatory gene profile in GM-MØ. **(A)** Schematic representation of the experiments. Monocytes were untreated or exposed to 50 nM of PMX at the beginning of the 7-day macrophage differentiation process with GM-CSF, and the mRNA levels were determined at day 7 on GM-MØ. **(B)** Volcano plot of RNAseq results showing the PMX-induced gene expression changes in GM-MØ. The number of annotated genes whose expression is upregulated or downregulated in GM-MØ after 7 days of PMX treatment (adjusted *p* < 0.05) is shown. **(C, D)** GSEA (http://software.broadinstitute.org/gsea/index.jsp) on the ranked comparison of the transcriptome of PMX-GM-MØ *versus* untreated GM-MØ, using the Hallmarks v7 data set available at the web site. **(E, F)** RSEM expression values of the informative genes from the indicated GSEA enriched plot and found in the leading edge; adj*p* value is indicated. **(G)** GSEA on the ranked comparison of the transcriptome of PMX-GM-MØ *versus* untreated GM-MØ, using the genes significantly modulated by MTX in GM-MØ as data set. **(H)** Venn diagram comparing the genes differentially expressed by PMX in GM-MØ with the genes significantly altered by MTX in GM-MØ. **(I)** Summary of GSEA with the indicated gene set on the ranked comparison of the transcriptomes of MTX-GM-MØ *vs.* GM-MØ, and PMX-GM-MØ *vs.* GM-MØ. Circled area is proportional to the absolute value of the normalized enrichment score (NES), which is indicated inside the circles. The intensity of color increases with the enrichment of the gene signature (red, positive enrichment; blue, negative enrichment). False discovery rate (FDRq) is also indicated. The gene sets used were the Hallmarks (v7.2) data set available at the web site and the “SREBP-dependent gene set” data set (34). For panels G–I, the transcriptome data of MTX-treated GM-MØ have been previously described (21) and are available at GEO (https://www.ncbi.nlm.nih.gov/geo/) (GSE71253). PMX, pemetrexed; GM-CSF, granulocyte-macrophage colony-stimulating factor; GSEA, gene set enrichment analysis; MTX, methotrexate.

We have previously described that low-dose MTX modifies the transcriptome of proinflammatory TS^+^ GM-CSF-primed macrophages (GM-MØ) (21) (GSE71253). Since PMX also influences the expression of MTX-regulated genes (*CCL20*, *LIF*, *MET*, and *GDF15*) (21), we sought to determine the whole range of transcriptional changes induced by PMX in human macrophages. Comparison of the gene signatures of GM-CSF-primed monocyte-derived macrophages in the absence (GM-MØ) or presence (PMX-GM-MØ) of PMX (50 nM) (**Figure 1A**) revealed a large number of significant transcriptional differences (**Figure 1B**) and that the gene profile of PMX-GM-MØ was enriched in terms like “HALLMARK_INFLAMMATORY_RESPONSE” and “TNFA_VIA_NFκB_SIGNALING” GSEA gene sets (31) (**Figures 1C, I**), supporting the notion that the continuous presence of PMX determines the acquisition of a more proinflammatory profile. Actually, PMX enhanced expression of NFκB-target genes and MTX-regulated genes (**Figure 1E**) and, like MTX (21), potentiated the expression of p53 target genes (**Figures 1D, F**). Moreover, comparison of the gene signature of MTX-GM-MØ (GSE71253) and PMX-GM-MØ revealed a large overlap of the respective transcriptional effects of both antifolates (**Figures 1G–I** and **Supplementary Figure 1**), thus emphasizing that, like MTX, PMX promotes the acquisition of a more proinflammatory and p53-dependent gene signature in GM-MØ. Regarding differences between antifolates, MTX-treated macrophages exhibit a significant over-representation of SREBP-dependent genes, while this over-representation is not seen in PMX-treated macrophages (**Figure 1I**). This difference is relevant because SREBP activity has been recently connected to macrophage polarization and to the capacity of macrophages to acquire a reparative profile (35).

### Pemetrexed Treatment Promotes a Transcriptional State of Tolerance to Lipopolysaccharide in Human Macrophages: Involvement of the TLR4 Signaling Pathway

**Figure 2:**
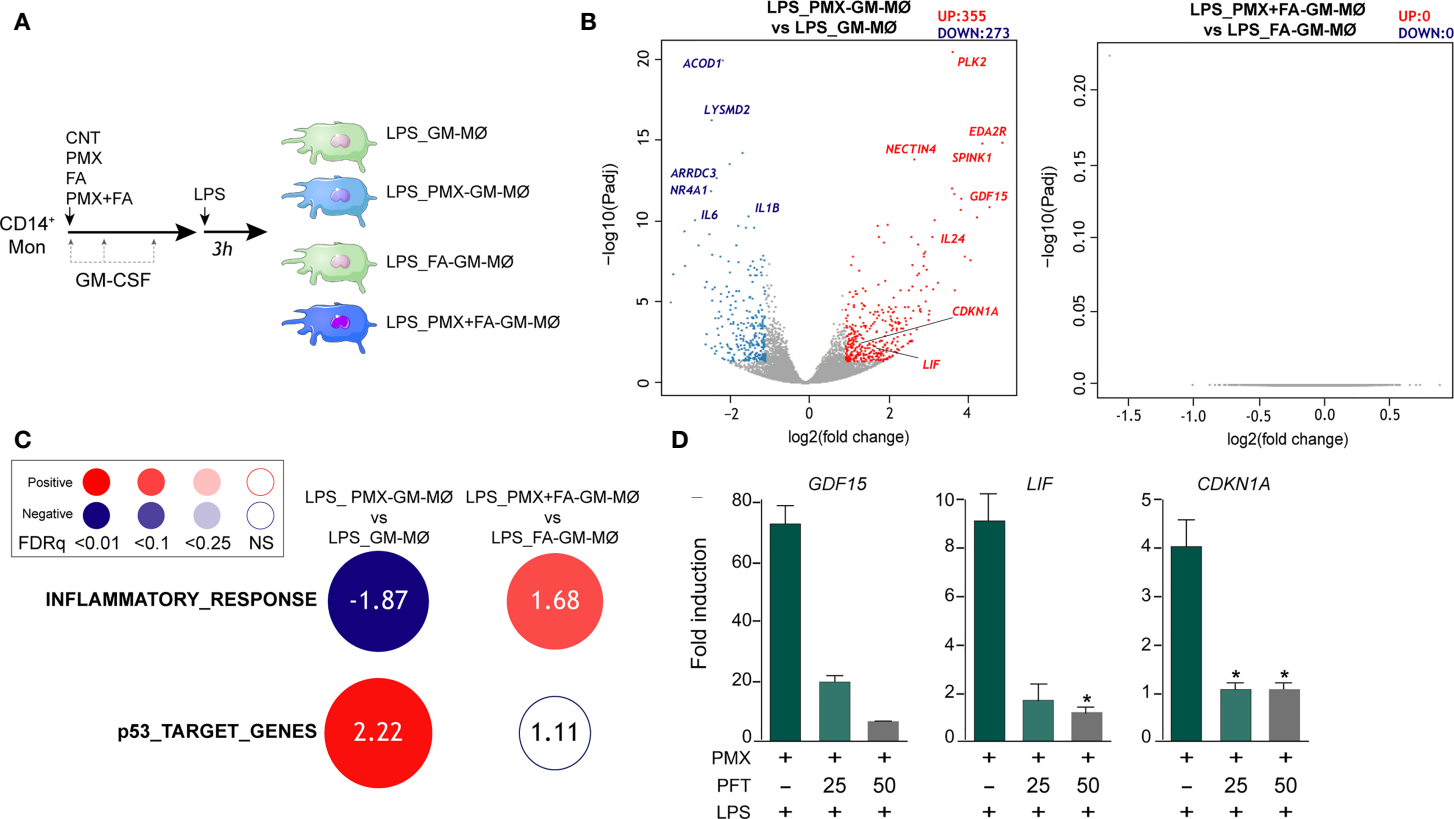
PMX promotes a transcriptional state of LPS tolerance. **(A)** Experimental design. Monocytes were untreated (GM-MØ), exposed to 50 nM of PMX (PMX-GM-MØ), 5 μM of folinic acid (FA-GM-MØ), or both (PMX+FA-GM-MØ) at the beginning of the 7-day macrophage differentiation process with GM-CSF and challenged with LPS (10 ng/ml) on day 7. Cells were assayed 3 h post LPS stimulation. **(B)** Volcano plot of RNAseq results showing gene expression changes 3 h post-LPS stimulation in PMX-treated GM-MØ (LPS_PMX-GM-MØ/LPS_GM-MØ, left) and PMX+FA-treated GM-MØ (LPS_PMX+FA-GM-MØ/LPS_FA-GM-MØ, right). The number of annotated genes whose expression is upregulated or downregulated 3 h post-LPS stimulation in GM-MØ after 7 days of PMX, FA, or PMX+FA treatment (adjusted *p* < 0.05) is shown. **(C)** Summary of GSEA with the indicated gene sets on the ranked comparison of the transcriptomes of LPS-treated PMX-GM-MØ *vs.* LPS-treated GM-MØ, and LPS-treated PMX+FA-GM-MØ *vs.* LPS-treated FA-GM-MØ. Circled area is proportional to the absolute value of the normalized enrichment score (NES), which is indicated inside the circles. The intensity of color increases with the enrichment of the gene signature (red, positive enrichment; blue, negative enrichment). False discovery rate (FDRq) is also indicated. The gene sets used were the Hallmarks v7.2 data set available at the web site and the “p53_Target_Gene” data set (32). **(D)** Gene expression determined by qRT-PCR on monocytes stimulated with PMX in the absence or presence of pifithrin-α (PFT, 25-50 μM) during the GM-CSF-dependent differentiation process and challenged with LPS for 3 h. Mean ± SEM of four independent donors are shown (^*^p < 0.05). Results are expressed as fold induction, which indicates the expression of each gene in PMX-exposed relative to control cells, and untreated or treated with PFT. PMX, pemetrexed; LPS, lipopolysaccharide; GSEA, gene set enrichment analysis.

Macrophage response to a given stimulus is dependent on extracellular cues that they have been previously exposed to (training/tolerance) (13–15). Thus, we next asked whether PMX promoted macrophage tolerance state at the transcriptional level. To that end, we determined the gene signature of LPS-stimulated (3 h) GM-MØ differentiated in the presence of PMX, FA (a reduced folate with high-affinity for RFC (36)], or PMX+FA (**Figure 2A**). Compared with LPS-treated GM-MØ, LPS-treated PMX-GM-MØ exhibited a significantly (|log_2_FC| >1; adj*p* < 0.05) elevated expression of 355 genes and diminished mRNA levels of 273 genes (**Figure 2B**). Supporting the specificity of PMX, none of these changes were observed in LPS-treated PMX+FA-GM-MØ, whose transcriptome was indistinguishable from that of control FA-GM-MØ (**Figure 2B**). Interestingly, GSEA revealed a very significant reduction of the genes within the GSEA “HALLMARK_INFLAMMATORY_RESPONSE” gene set (31) (**Figure 2C**) in LPS-treated PMX-GM-MØ, a negative enrichment that was not seen in PMX+FA-GM-MØ (**Figure 2C**). Therefore, the presence of PMX during macrophage differentiation impairs the LPS-induced expression of inflammation-related genes, thus indicating that PMX promotes the acquisition of a tolerance state in macrophages. Conversely, expression of the “p53_Target_Genes” gene set was significantly and specifically enriched in LPS-treated PMX-GM-MØ (**Figure 2C**). Moreover, the role of p53 in the PMX-induced tolerance was suggested by using the p53 inhibitor pifithrin-α, which dose dependently diminished the expression of three previously reported PMX-responsiveness genes (*GDF15*, *LIF*, and *CDKN1A* mRNA) in LPS-treated PMX-GM-MØ (**Figure 2D**). Since the *CDKN1A*-encoded p53 target gene p21 determines the reparative and anti-inflammatory profile of macrophages (37), this result supports the notion that PMX conditions macrophages towards a tolerance transcriptional state.

**Figure 3:**
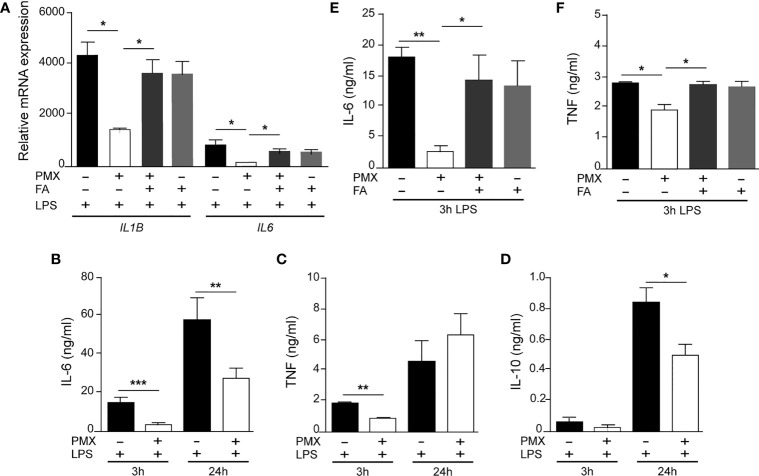
PMX alters TLR4 responsiveness in GM-CSF-primed macrophages. **(A)** Expression of *IL1B* and *IL6* by monocytes differentiated with GM-CSF in the absence or presence of PMX (50 nM), folinic acid (FA; 5 µM), or both and challenged with LPS for 3 h, as determined by qRT-PCR. Mean ± SEM of three independent donors are shown. Groups were compared by applying one-way ANOVA (^*^p < 0.05). Production of IL-6 **(B)**, TNFα **(C)**, or IL-10 **(D)** by monocytes differentiated with GM-CSF in the absence or presence of PMX and challenged with LPS for 3 and 24 h, as determined by ELISA. Mean ± SEM of seven independent donors are shown (^*^p < 0.05, ^**^p < 0.01, ^***^p < 0.001). Production of IL-6 **(E)** and TNFα **(F)** by monocytes differentiated with GM-CSF in the absence or presence of PMX (50 nM), folinic acid (FA; 5 µM), or both and challenged with LPS for 3 h, as determined by ELISA. Mean ± SEM of five independent donors are shown. Groups were compared by applying one-way ANOVA (with Tukey’s post-hoc test, ^*^p < 0.05, ^**^p < 0.01). PMX, pemetrexed; GM-CSF, granulocyte-macrophage colony-stimulating factor; LPS, lipopolysaccharide.

To evaluate the functional correlate of the PMX-induced transcriptional tolerance, we analyzed the ability of PMX to modify human macrophage responses upon TLR4 engagement. In agreement with the RNAseq data, LPS-treated (3 h) PMX-GM-MØ displayed a considerably diminished *IL6* and *IL1B* mRNA expression when compared with LPS-treated GM-MØ (**Figure 3A**). Moreover, PMX-GM-MØ produced significantly lower levels of LPS-induced IL-6, TNFα, and IL-10 after both short-term (3 h) and long-term (24 h, for IL-6 and IL-10) LPS exposure (**Figures 3B–D**). The specificity of the PMX effect was demonstrated by the absence of the inhibitory effect in PMX+FA-GM-MØ (shown in **Figures 3A, E, F**), thus indicating that the effects of PMX are mediated through OCM blockade. Of note, PMX-GM-MØ also produced lower level of IL-6 and IL-10 after exposure to the TLR2 ligand lipoteichoic acid (LTA), thus indicating that PMX attenuates inflammatory cytokine expression in response to either TLR4 or TLR2 ligands (**Supplementary Figure 2**). Altogether, these results indicate that PMX conditions macrophages for the acquisition of a tolerance state at the transcriptional and functional levels.

**Figure 4:**
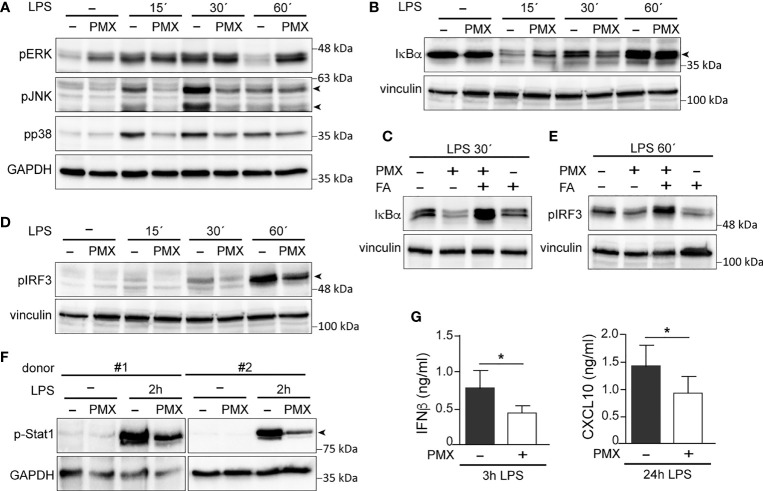
PMX alters TLR4 signaling in GM-CSF-primed macrophages. **(A, B)** Immunoblot analysis of pERK, pJNK, and pp38 **(A)** and IκBα **(B)** by monocytes differentiated with GM-CSF in the absence or presence of PMX for 7 days and challenged with LPS for the indicated time points. **(C)** Immunoblot analysis of IκBα by monocytes differentiated with GM-CSF in the absence or presence of PMX, PMX+FA or FA for 7 days and challenged with LPS for 30 min. **(D, E)** Immunoblot analysis of pIRF3 by monocytes differentiated with GM-CSF in the absence or presence of PMX **(D)**, folinic acid (FA), or both **(E)** for 7 days and challenged with LPS for the indicated time points. **(F)** Immunoblot analysis of pStat1 by two independent preparations of monocytes differentiated with GM-CSF in the absence or presence of PMX for 7 days and challenged with LPS for 2 h. In panels **(A–F)**, a representative experiment of two to four independent donors is shown. Molecular weight markers are indicated. Arrowheads indicate the protein of interest. **(G)** Production of IFNβ and CXCL10 by monocytes differentiated with GM-CSF in the absence or presence of PMX challenged with LPS for 3 h (IFNβ) or 24 h (CXCL10), as determined by ELISA. Mean ± SEM of seven independent donors are shown (^*^p < 0.05). PMX, pemetrexed; GM-CSF, granulocyte-macrophage colony-stimulating factor; LPS, lipopolysaccharide.

Previous studies have demonstrated that tolerant-macrophages exhibit a lower level of MAPK and NFκB activation upon TLR stimulation (22, 38, 39). To determine whether PMX promotes “bona fide” innate tolerance in macrophages, we first determined the levels of MAPK and IκBα activation in LPS-treated PMX-GM-MØ. Upon LPS stimulation, PMX-treated GM-MØ exhibited a reduced activation of p38 and JNK compared with untreated GM-MØ (**Figure 4A**). By contrast, and as described in other cellular systems (40), PMX-GM-MØ showed an increased and more sustained phosphorylation of ERK1/2 in response to LPS (**Figure 4A**). Besides, a slower kinetics of IκBα degradation was detected in LPS-exposed PMX-GM-MØ (**Figure 4B**), an effect abolished in the presence of FA (PMX-FA-GM-MØ, **Figure 4C**). Further and compared with GM-MØ, LPS induced lower levels of p-IRF3, p-STAT1, and IFNβ1 and CXCL10 secretion in PMX-GM-MØ (**Figures 4D–G**). Taken together, these results demonstrate that OCM blockade impairs TLR4-initiated intracellular signaling and the establishment of a state of LPS tolerance.

### Pemetrexed and Methotrexate Regulate CD14 Expression in Granulocyte-Macrophage Colony-Stimulating Factor-Primed Human Macrophage

**Figure 5:**
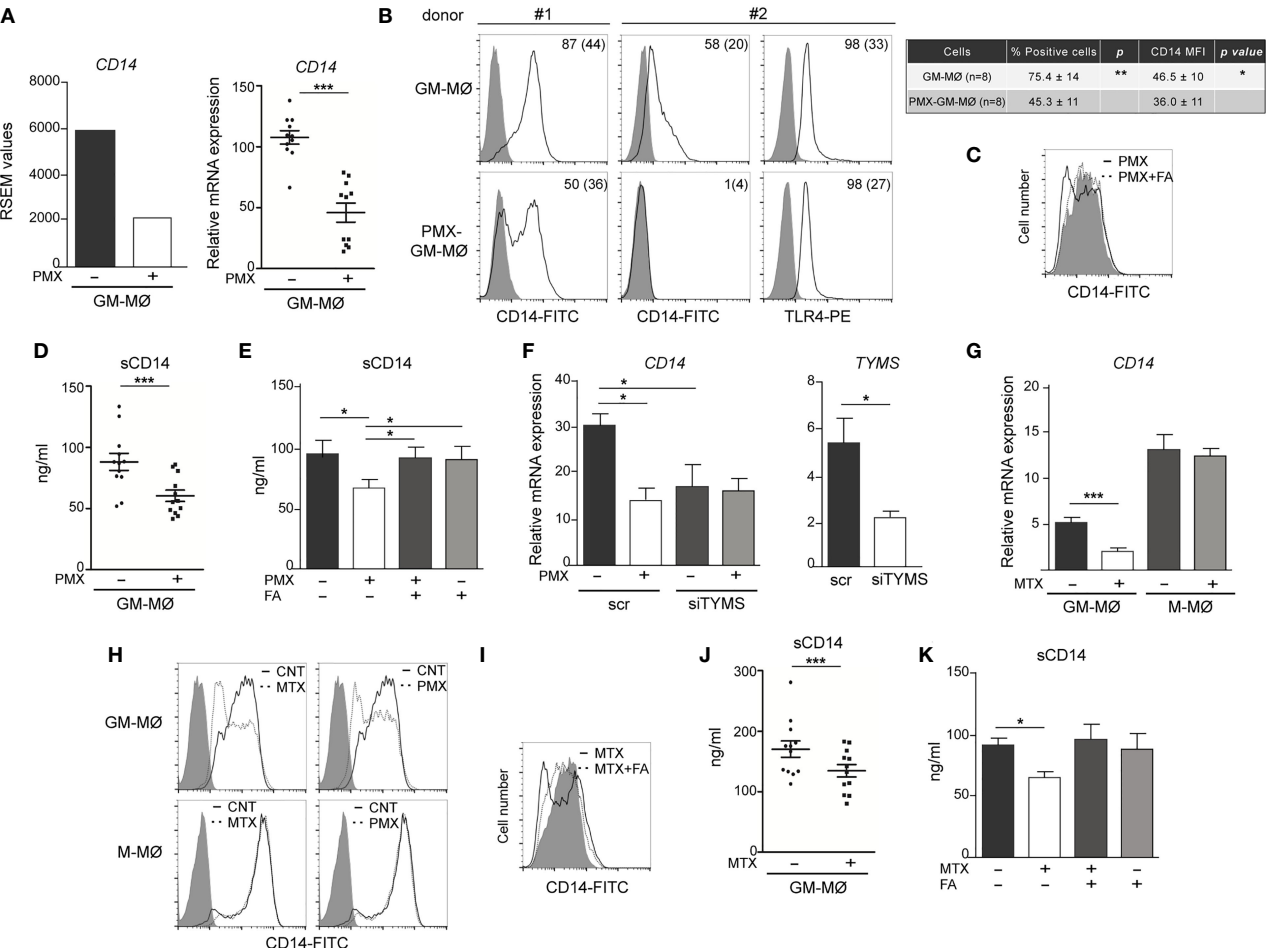
OCM blockade diminished CD14 expression on GM-CSF-primed macrophages. **(A)** *CD14* mRNA expression on monocytes stimulated with PMX during the GM-CSF-dependent differentiation process, as determined by RNAseq (adj*p* = 10^−5^, left) and by qRT-PCR (right, n = 11 independent donors; each symbol represents a single donor, ^***^p < 0.001). **(B)** Cell surface expression of CD14 and TLR4 on monocytes differentiated with GM-CSF in the absence or in the presence of PMX, as determined by flow cytometry. Filled histograms indicate the fluorescence levels produced by an isotype-matched antibody. The percentage of marker-positive cells and the mean fluorescence intensity (in parentheses) are indicated. The experiment was done on eight independent donors, and two representative donors are shown. The average of percentage of positive cells and MFI ± SD for CD14 in GM-MØ and PMX-GM-MØ is shown in the right panel. Groups were compared by applying the t-test (^*^p < 0.05, ^**^p < 0.01). **(C)** Cell surface expression of CD14 on monocytes differentiated with GM-CSF in the absence (filled histograms) or in the presence of PMX (empty histograms) or PMX+FA for 7 days (histograms with a dotted line), as determined by flow cytometry. The experiment was done on four independent donors, and a representative is shown. **(D)** Soluble CD14 level in the supernatant of monocytes differentiated with GM-CSF in the absence or in the presence of PMX for 7 days, as determined by ELISA (n = 12 independent donors; each symbol represent a single donor; ^***^p < 0.001). **(E)** Soluble CD14 level in the supernatant of monocytes differentiated with GM-CSF in the absence or presence of PMX, folinic acid (FA), or both, as determined by ELISA (n = 6 independent donors). Groups were compared by applying one-way ANOVA (with Tukey’s post-hoc test, ^*^p < 0.05). **(F)** Left, *CD14* mRNA expression on GM-MØ transfected with control siRNA (scr) and siRNA for thymidylate synthase (siTYMS) and exposed to PMX for 48 h, as determined by qRT-PCR (n = 4 independent donors). Groups were compared by applying one-way ANOVA (with Tukey’s post-hoc test, ^*^p < 0.05). Right, *TYMS* mRNA expression on GM-MØ transfected with control siRNA and siRNA for TYMS for 48 h, as determined by qRT-PCR (n = 4 independent donors, ^*^p < 0.05). **(G)** *CD14* mRNA expression on monocytes stimulated with MTX (50 nM) during the GM-CSF or M-CSF-dependent differentiation process, as determined by qRT-PCR (n = 6 independent donors, ^***^p < 0.001). **(H)** Cell surface expression of CD14 on monocytes differentiated with GM-CSF (GM-MØ) or M-CSF (GM-MØ) in the absence (empty histograms) or in the presence of MTX or PMX (histograms with a dotted line), as determined by flow cytometry. Filled histograms indicate the fluorescence levels produced by an isotype-matched antibody. **(I)** Cell surface expression of CD14 on monocytes differentiated with GM-CSF in the absence (filled histograms) or in the presence of MTX (empty histograms) or MTX+FA (histograms with a dotted line), as determined by flow cytometry. For panels H and I, the experiment was done on four independent donors, and a representative is shown. **(J)** Soluble CD14 level in the supernatant of monocytes differentiated with GM-CSF in the absence or in the presence of MTX for 7 days, as determined by ELISA (n = 15 independent donors, each symbol represent a single donor; ^***^p < 0.001). **(K)** Soluble CD14 level in the supernatant of monocytes differentiated with GM-CSF in the absence or presence of MTX, folinic acid (FA), or both, as determined by ELISA (n = 4 independent donors). Groups were compared by applying one-way ANOVA (with Tukey’s post-hoc test, ^*^p < 0.05). OCM, one-carbon metabolism; GM-CSF, granulocyte-macrophage colony-stimulating factor; PMX, pemetrexed; MFI, mean fluorescence intensity.

Next, we determined whether PMX affected the expression of negative regulators of TLR-induced cytokine production that contribute to LPS tolerance (38). Unlike MTX-induced tolerance, mostly mediated by increased *TNFAIP3* (A20) expression (38, 39, 41, 42), no difference in *TNFAIP3* expression was found between LPS-treated GM-MØ and LPS-treated PMX-GM-MØ (not shown). Thus, we next screened for genes coding for regulators of TLR-induced activation and whose expression significantly differs between GM-MØ and PMX-GM-MØ, and we found that PMX-GM-MØ cells express significantly lower levels of *CD14* mRNA than GM-MØ (**Figure 5A**). After validation of this result in additional samples (shown in **Figure 5A**), we confirmed that PMX-GM-MØ cells express a significantly lower level of cell surface CD14 [membrane-bound CD14 (mCD14)] than GM-MØ (**Figures 5B, C**). Specifically, the percentage of CD14-positive cells was 75% in GM-MØ and 45% in PMX-GM-MØ, whereas the mean fluorescence significantly varied at 46.5 and 36, respectively (**Figure 5B**). Further, sCD14 was also significantly lower in the supernatant of PMX-GM-MØ than in control GM-MØ cells (**Figure 5D**), and the PMX-induced reduction of mCD14 and sCD14 was completely reversed in the presence of FA (**Figures 5C, E**). Of note, *TS* knockdown diminished CD14 expression without further effect of PMX (**Figure 5F**), thus indicating that the PMX-triggered CD14 downregulation is dependent on TS expression. Of note, the antifolate MTX exhibited the same effect, as MTX-GM-MØ showed significantly lower expression of *CD14* mRNA, mCD14, and sCD14 than did GM-MØ, an effect also prevented by FA (**Figures 5G–K**). Therefore, OCM blockade mediates the loss of *CD14* gene and protein expression in macrophages differentiated in the presence of antifolates (PMX-GM-MØ or MTX-GM-MØ). The physiological significance of these findings was further stressed by the fact that both antifolates only impaired CD14 expression in proinflammatory GM-MØ, whereas they did not modify CD14 expression in M-CSF-conditioned macrophages (M-MØ) (**Figures 5G, H**), which resemble anti-inflammatory tissue-resident and pro-tumoral TAM (20, 43, 44).

### Exogenous Soluble CD14 Restores the Impaired Lipopolysaccharide Responsiveness of Pemetrexed or Methotrexate-GM-MØ

**Figure 6:**
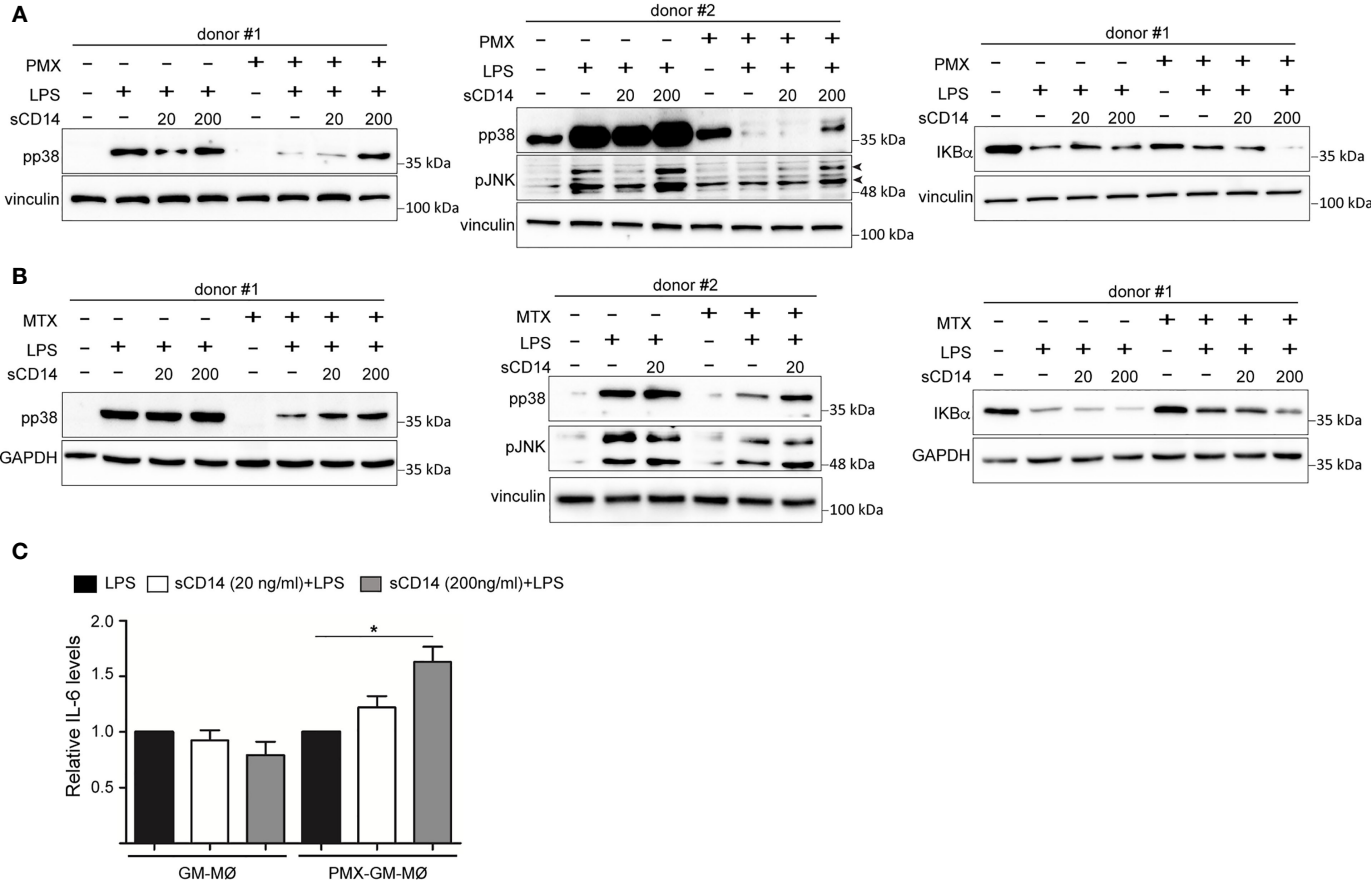
sCD14 restores p38 and JNK activation and IL-6 production in LPS-treated PMX-GM-MØ. Immunoblot analysis of pp38, pJNK, and IκBα by GM-MØ and PMX-GM-MØ **(A)** or MTX-GM-MØ **(B)** challenged with LPS in the absence or presence of 20 ng/ml (20) or 200 ng/ml (200) of sCD14 for 15 min (pp38, pJNK) and 30 min (IκBα). The experiment was performed in four independent donors, and two of them are shown. The signal in pp38 in donor #2 **(A)** is saturated for better detection of lane 8 (sCD14 200) in PMX-GM-MØ. Molecular weight markers are indicated. Arrowheads indicate the protein of interest. **(C)** Production of IL-6 by GM-MØ or PMX-GM-MØ challenged with LPS in the absence or presence of 20 or 200 ng/ml of sCD14 for 3 h, as determined by ELISA. The experiment was performed in four independent donors, and the relative production of IL-6 is shown (*p < 0.05). LPS, lipopolysaccharide; PMX, pemetrexed; sCD14, soluble CD14.

Since the reduced expression of sCD14 and mCD14 correlated with diminished intracellular signaling and cytokine responses to LPS in PMX-GM-MØ, we next determined whether CD14 had a role in the PMX-induced macrophage tolerance state by assaying LPS responsiveness of PMX-GM-MØ in the presence of increasing concentrations of exogenous sCD14. Addition of sCD14 increased the LPS-induced activation of p38 and JNK, as well as the LPS-induced degradation of IκBα in PMX-GM-MØ, but not in GM-MØ (**Figure 6A**). Noteworthy, the same results were found in MTX-GM-MØ (**Figure 6B**). Moreover, sCD14 dose dependently increased the LPS-induced IL-6 production in PMX-GM-MØ, but not in LPS-treated GM-MØ (**Figure 6C**). Therefore, exogenous sCD14 partly restores the inhibitory effect of PMX on TLR4-initiated intracellular signaling and IL-6 production and demonstrates that CD14 mediates the OCM-dependent pro-tolerant effect of PMX.

### Soluble CD14 Diminishes in Early Rheumatoid Arthritis Patients Who Are Methotrexate Responders

**Figure 7:**
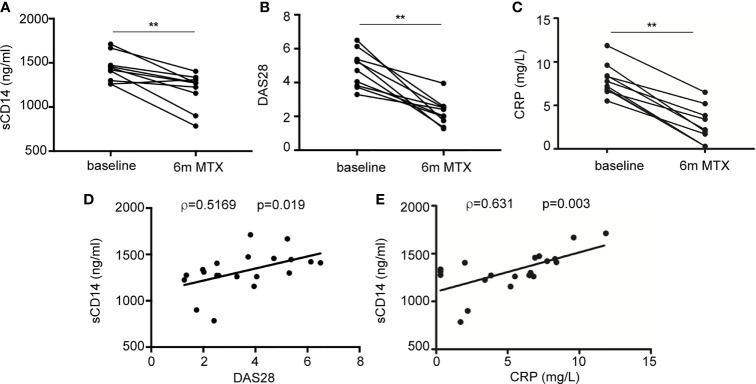
Plasma level of sCD14 decreases in RA patients who are MTX responders. **(A)** sCD14 level in plasma from early RA patients at baseline and 6 months after initiating MTX treatment (15–25 mg/week), as determined by ELISA (n = 10, ^**^p < 0.01, Wilcoxon test). Disease activity score (DAS28) **(B)** and C-reactive protein (CRP) **(C)** from early RA patients at baseline and 6 months after MTX treatment from the discovery cohort. Correlation between sCD14 and DAS28 **(D)** sCD14 and CRP **(E)** in early RA patients (two-tailed Spearman’s correlation). RA, rheumatoid arthritis; MTX, methotrexate; sCD14, soluble CD14.

**Figure 8:**
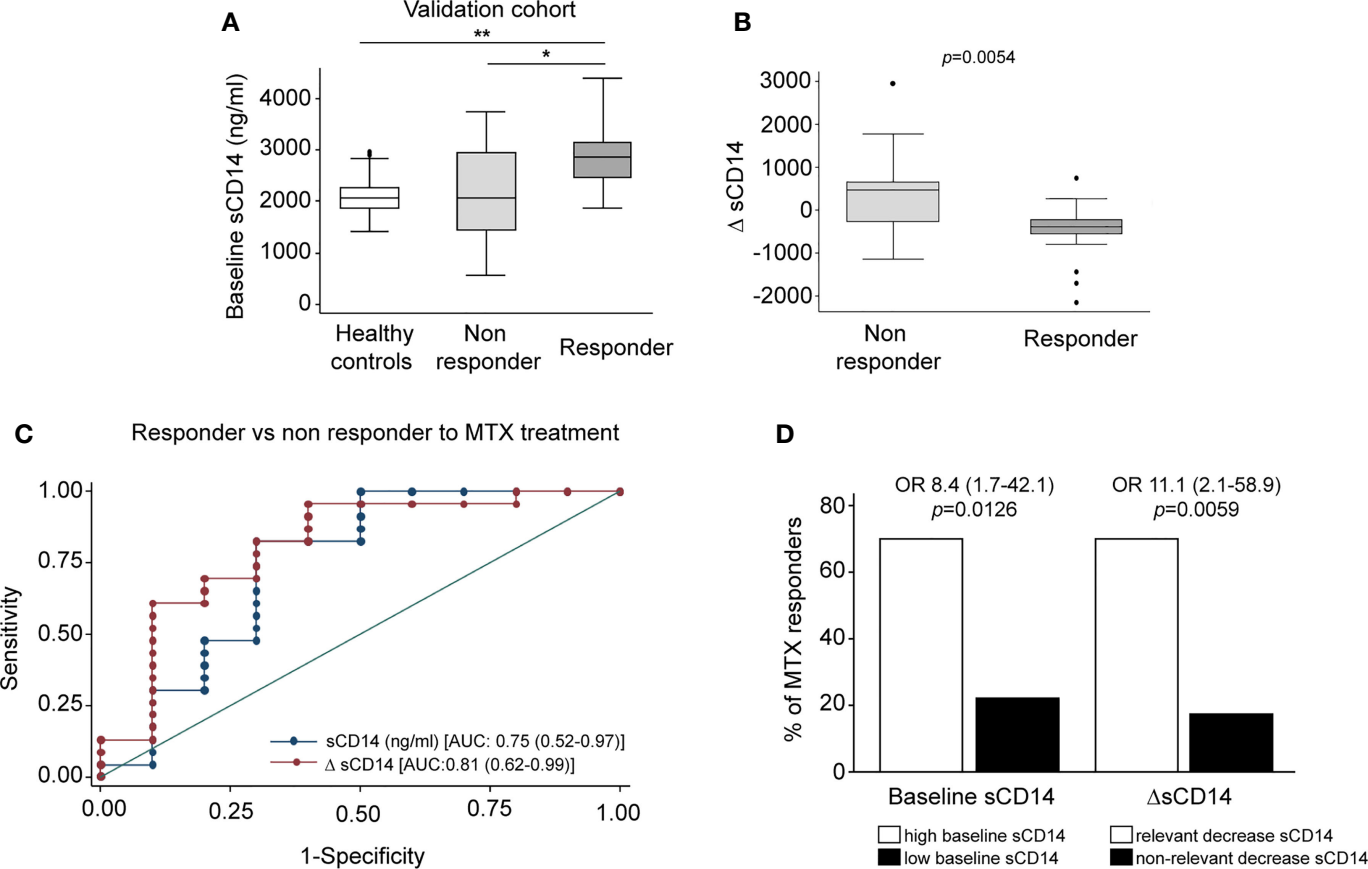
Serum level of sCD14 decreases in RA patients who are MTX responders in a validation cohort. **(A)** Baseline sCD14 in healthy donors (white box, n = 40), MTX-non-responder (soft gray box, n = 10), and MTX-responder (dark gray box, n = 23) RA patients. Statistical significance respect to MTX-responders was determined through Mann–Whitney test (*p < 0.05; **p < 0.01). **(B)** Variation in sCD14 between baseline and 6 months’ follow-up visits (ΔsCD14: sCD14 value in 6-month visit − sCD14 value at baseline visit) in MTX-non-responder (soft gray box) and MTX-responder (dark gray box) RA patients. Data in panels A and B are shown as interquartile range (p75 upper edge of box, p25 lower edge, and p50 midline) as well as the p95 (line above box) and p5 (line below). **(C)** Receiver operating characteristic curve analysis to assess capacity of baseline sCD14 (blue line and dots) and ΔsCD14 (red line and dots) to discriminate between MTX-responder and MTX-non-responder RA patients. **(D)** Comparison of the percentage of MTX responders in patients with high (left white bar) versus low (left black bar) baseline sCD14 and in patients with relevant decrease of sCD14 (right white bar) versus non-relevant variation of sCD14 (right black bar). The cutoff for discriminating high and low sCD14 was 2,460 ng/ml, and the one to discriminate between relevant and not relevant decrease of sCD14 was 188 ng/ml, as described in the last section of *Results*. OR and its CI were estimated with the cs command of Stata 14.1 and the significance level with Fisher’s test. sCD14, soluble CD14; RA, rheumatoid arthritis; MTX, methotrexate.

Finally, we sought to determine the therapeutic relevance of the CD14 downregulation in GM-MØ generated in the presence of antifolates. Although PMX and MTX similarly diminished mCD14 and sCD14 in GM-MØ (shown in **Figure 5**), only low-dose MTX is an anchor drug for RA treatment (23, 45). Therefore, we turned to RA patients treated with MTX to evaluate the clinical significance of the association between antifolate exposure and CD14 downregulation. Specifically, we determined sCD14 level in plasma from MTX-responder RA patients both at baseline and after 6 months of treatment with MTX. We found that sCD14 levels significantly decreased in patients who respond to MTX (**Figure 7A**). Moreover, sCD14 levels positively correlated with disease activity score DAS28 and CRP (**Figures 7B–E**). These results indicate that sCD14 expression is lower in MTX responder RA patients, suggesting that sCD14 might be a biomarker for MTX response. To confirm these findings, we determined sCD14 concentration in a validation cohort of early arthritis patients at baseline and during 6 months of MTX monotherapy, including both MTX-responder and non-responder patients (see *Materials and Methods* for definition). Interestingly, baseline sCD14 levels in MTX-non-responder patients were similar to those in control healthy donors, whereas MTX-responder patients exhibited significantly higher sCD14 serum levels (**Figure 8A**). Moreover, sCD14 levels diminished in MTX-responder patients but not in MTX non-responders (**Figure 8B**). In order to determine whether baseline sCD14 and ΔsCD14 could be MTX response biomarkers, we performed ROC analysis. Although the area under the curve (AUC) of ΔsCD14 was slightly higher than that of baseline sCD14 (**Figure 8C**), it did not reach statistical significance. The best cutoff to discriminate between MTX responder and non-responder patients was 2,460 ng/ml for baseline sCD14 (78% sensitivity and 70% specificity) and 188 ng/ml for ΔsCD14 (83% sensitivity and 70% specificity). The OR for having high baseline sCD14 and being MTX responder was 8.4 (*p* = 0.0126), whereas the OR for a decreased sCD14 was 11.1 (*p* = 0.0059) (**Figure 8D**). Furthermore, after adjustment by variables related to MTX response such as age, gender, and baseline DAS28, the OR increased to 25.5 (for sCD14) and 40.35 (for ΔsCD14) (**Supplementary Table 1**). Altogether, these results indicate that determination of sCD14 levels could be a valuable tool to predict or evaluate MTX response in RA patients.

## Discussion

OCM is a complex network of biosynthetic pathways that includes *de novo* biosynthesis of purines and thymidylate, amino acid metabolism, and methylation reactions (1). In the present report, we describe the impact of OCM on the functional and gene expression profile of GM-CSF-primed human monocyte-derived macrophages (GM-MØ). We have found that OCM blocker PMX induces the acquisition of a more proinflammatory and p53-dependent gene signature in human macrophages, an effect that resembles the transcriptomic changes triggered by MTX, another antifolate with OCM-blocking activity (**Figure 1** and **Supplementary Figure 1**) (21). Moreover, we describe that blocking OCM reprograms GM-MØ towards a tolerance state, as PMX-GM-MØ cells exhibit a reduced response to TLR ligands. LPS was used as a secondary stimulus because several TLR4 ligands have been detected in the synovial fluid of patients with active RA and also because low doses of LPS are commonly used to exemplify cross-tolerance in myeloid cells. The specificity of PMX effects was demonstrated by the absence of the inhibitory effect in the presence of FA, a reduced folate with high affinity for RFC (36). Mechanistically, PMX reduces LPS-induced p38, JNK, IRF3, and STAT1 activation; IκBα degradation; and inflammatory cytokine production in human macrophages. In line with these findings, PMX also diminished the expression of mCD14 and sCD14, a co-receptor for TLR4 required for MyD88-dependent signaling at low concentrations of LPS, and for LPS-induced TLR4 internalization into endosomes and activation of TRIF-mediated signaling (46, 47). Accordingly, PMX-GM-MØ cells exhibit diminished MyD88-dependent and TRIF-mediated signaling, as well as reduced cytokine production (IL-6 and IFNβ1), upon exposure to LPS. The relevance of sCD14 in the OCM-dependent pro-tolerant effect of PMX is supported by the ability of exogenous sCD14 to restore the LPS sensitivity of PMX-GM-MØ. Altogether, these results indicate that the global anti-inflammatory activity of PMX relies on its ability to induce a proinflammatory profile in GM-CSF-primed macrophages, making PMX-conditioned macrophages less responsive to secondary inflammatory stimuli. These results link OCM to innate immune tolerance (48) and demonstrate that antifolates promote a proinflammatory state in macrophages and, in parallel, trigger a loss of CD14 expression, all of which end up establishing a tolerant state and an impaired response to subsequent stimulation (LPS and LTA).

Cellular metabolism is a critical mediator of the reprogramming of myeloid cells that takes place during trained immunity (49). Increased aerobic glycolysis is a hallmark of β-glucan or Bacillus Calmette–Guérin (BCG)-induced trained immunity in monocytes (50, 51). Regarding innate tolerance, the metabolite itaconate inhibits LPS-mediated IκB induction and induces tolerance in human monocytes (52). The lipid and amino acid metabolisms are also important for the induction of trained immunity (49), as metabolites of the cholesterol synthesis pathway are crucial for establishing β-glucan-, BCG-, or oxidized low-density lipoprotein (oxLDL)-induced trained immunity in macrophages (53). We now describe that exposure of human macrophages to antifolates (PMX or MTX) results in the acquisition of an innate tolerance state, thus demonstrating that the OCM is another metabolic circuit that critically mediates trained immunity.

CD14 acts both as a pattern recognition receptor (54, 55) and as a receptor for LPS (56). In the context of RA, Lewis et al. have recently defined three distinct histopathological entities based on transcriptional data from the synovium of early treatment-naive RA patients: fibroblastic pathotype, macrophage-rich myeloid pathotype, and lympho-myeloid pathotype (57). The analysis of CD14 mRNA expression in the three pathotypes (https://peac.hpc.qmul.ac.uk/) revealed that CD14 expression is higher in lymphoid pathotype (adj*p* 5.7e−0^3^ versus myeloid pathotype, adj*p* 6.8e−0^7^ versus fibroid pathotype), indicating that CD14 marks the lymphoid-rich pathotype with high plasma cell accumulation. Moreover, synovium CD14 expression correlated positively with disease activity (adj*p* 0.016) (57, 58). Along the same line, sCD14 levels are increased in RA synovial fluid and serum compared with osteoarthritis patients (59, 60). sCD14 plays an important role in mediating the immune responses to LPS of CD14-negative cells such as endothelial cells and epithelial cells and induces proinflammatory cytokines in fibroblast-like synovial cells from RA patients (61). The modulation of CD14 expression by antifolates that we now report supports the anti-inflammatory role of MTX in RA patients and leads us to suggest that MTX-treated RA patients would exhibit lower levels of sCD14 and mCD14 in myeloid cells, lower responsiveness to TLR4-dependent damage-associated molecular patterns (DAMPs), and a lower proinflammatory profile. In line with this hypothesis, we have observed that sCD14 level diminishes in serum of early RA patients responding to MTX treatment. Considering that MTX is the first line in RA treatment and the importance of taking advantage of the window of opportunity to achieve early remission, it would be of interest to select those patients with the highest odds to be MTX responders. In this regard, a low baseline sCD14 can identify patients who are MTX non-responders, in order to try a DMARD with other mechanism of action. We are aware that there is enough overlap of sCD14 baseline levels between responder and non-responder patients. In this case, it would be also useful to measure the variation in sCD14 levels that could help to better identify non-responder patients in doubtful cases. We acknowledge that a validation cohort with a larger number of patients should be analyzed in the future to gain more robust results.

On the other hand, it is well known that MTX withdrawal due to adverse events is more frequent than inefficacy (62). PMX is a chemotherapeutic drug with substantial activity against lung carcinomas usually considered as refractory to classical antifolates (63). Although not used as a DMARD in RA, PMX has been shown to suppress the release of TNFα from activated T cells of RA patients and also ameliorates experimental arthritis in a model of collagen-induced arthritis in rats, thus indicating that PMX exhibits an anti-inflammatory action both *ex vivo* and *in vivo* (12, 64). In spite of these antecedents, the anti-inflammatory efficacy of PMX has not been previously tested in the case of innate immune cells. Our results indicate that besides a robust antifolate-mediated cytostatic effect, PMX exerts a huge reprogramming effect on human macrophages, thus opening further research of this drug to new opportunities beyond the limit of its actual clinical utility.

## Data Availability Statement

The original contributions presented in the study are publicly available. These data can be found here: https://www.ncbi.nlm.nih.gov/geo/query/acc.cgi?acc=GSE159380 and GSE159380.

## Ethics Statement

The studies involving human participants were reviewed and approved by Hospital La Princesa Ethics Committee (PI-518, March 28, 2011). The patients/participants provided their written informed consent to participate in this study.

## Author Contributions

SF-R, CB-A, RG, MT, IM, and AT-M designed the research, performed the research, and analyzed the data. LN, AV, RG-V, GJ, M-EM-C, and IG-A designed the research and analyzed the data. AP-K conceived the study, designed the research, analyzed the data, and wrote the paper. All authors contributed to the article and approved the submitted version.

## Funding

This work was supported by grants PI17/00037 and PI20/00316 to AP-K; PI18/00371 to IG-A; and RIER RD16/0012/0007, RD16/0012/0011, and RD16/0012/0012 from Instituto de Salud Carlos III/FEDER to AP-K, M-EM-C, and IG-A and cofinanced by European Regional Development Fund “A way to achieve Europe” (ERDF). SF-R is supported by a contract from Instituto de Salud Carlos III (FI18/00109), and AT-M is supported by a PhD fellowship from the Autonomous Region of Madrid (PEJD-2019-PRE/BMB-16851).

## Conflict of Interest

IG-A reports personal fees from Lilly and Sanofi; personal fees and non-financial support from BMS; personal fees and non-financial support from AbbVie; research support, personal fees, and non-financial support from Roche Laboratories; and non-financial support from MSD, Pfizer, and Novartis, not related to the submitted work.

The remaining author declares that the research was conducted in the absence of any commercial or financial relationships that could be construed as a potential conflict of interest.

## Publisher’s Note

All claims expressed in this article are solely those of the authors and do not necessarily represent those of their affiliated organizations, or those of the publisher, the editors and the reviewers. Any product that may be evaluated in this article, or claim that may be made by its manufacturer, is not guaranteed or endorsed by the publisher.
