# Supplementary material for: The Macrophage Reprogramming Ability of Antifolates Reveals Soluble CD14 as a Potential Biomarker for Methotrexate Response in Rheumatoid Arthritis
Source: Front Immunol. 2021 Nov 5;12:776879. doi: 10.3389/fimmu.2021.776879 (PMC8602851; doi:10.3389/fimmu.2021.776879)
Supplement: Supplementary file 1 [file DataSheet_1.pdf]

Supplementary Figure 1

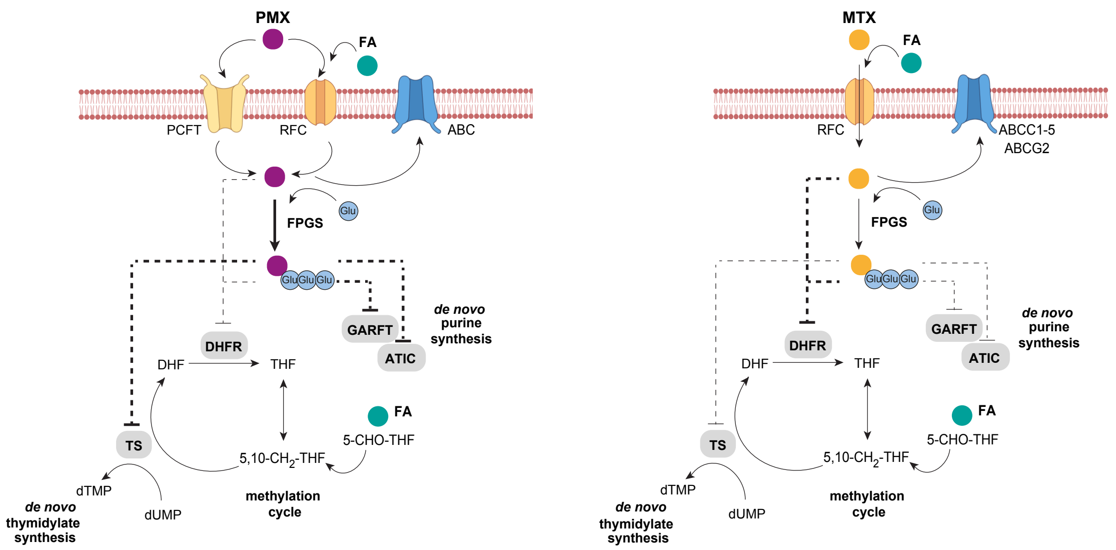

Supplementary Figure 1. Differences between pemetrexed (PMX) and methotrexate (MTX) in cellular uptake, polyglutamation and targeting folate-dependent enzymes. PMX (left) enters the cells through reduced folate carrier (RFC) and proton-coupled folate transporter (PCFT), whereas MTX (right) is transported into the cells via RFC. PMX is a better substrate for folylpoly-γ glutamate synthase (FPGS), that catalyzes the addition of glutamate residues to (anti)folates, than MTX. Polyglutamates of PMX are inhibitors of thymydylate synthase (TS), glycinamide ribonucleotide transferase (GARFT), dihydrofolate reductase (DHFR) and aminoimidazole-carboxamide ribonucleotide formyl transferase (ATIC), but PMX is primarly a TS inhibitor. MTX is a potent inhibitor of DHFR and polyglutamates of MTX also inhibit TS and GARFT. Folinic acid (5-formyl tetrahydrofolate, FA) is a reduced folate that enters the cells through RFC, and has a similar affinity for RFC than PMX and MTX. Biorender.com was used to create this Figure.
