# Supplementary material for: The Macrophage Reprogramming Ability of Antifolates Reveals Soluble CD14 as a Potential Biomarker for Methotrexate Response in Rheumatoid Arthritis
Source: Front Immunol. 2021 Nov 5;12:776879. doi: 10.3389/fimmu.2021.776879 (PMC8602851; doi:10.3389/fimmu.2021.776879)
Supplement: Supplementary file 2 [file DataSheet_2.pdf]

# Supplementary Figure 2

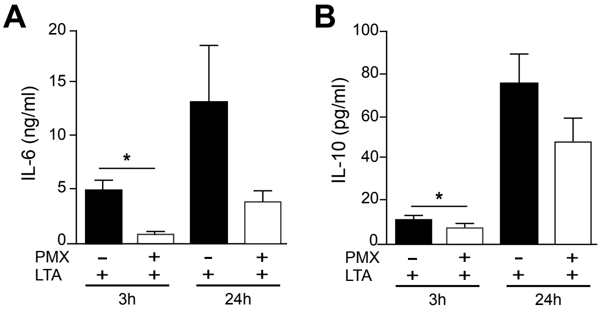


**Supplementary Figure 2.** Production of IL-6 and IL-10 by monocytes differentiated with GM-CSF in the absence or presence of PMX for 7 days and challenged with lipoteichoic acid (LTA) for 3h and 24h, as determined by ELISA. Mean ± SEM of 6 independent donors are shown (*p<0.05).
