# Supplementary material for: The Macrophage Reprogramming Ability of Antifolates Reveals Soluble CD14 as a Potential Biomarker for Methotrexate Response in Rheumatoid Arthritis
Source: Front Immunol. 2021 Nov 5;12:776879. doi: 10.3389/fimmu.2021.776879 (PMC8602851; doi:10.3389/fimmu.2021.776879)
Supplement: Supplementary file 3 [file DataSheet_3.pdf]

**High basal sCD14 (ng/ml) levels**		
	**Odds Ratio (95% CI)**	**p value**
**Female gender**	0.11 (0.005 to 2.37)	0,16
**Age (years)**	0.93 (0.85 to 1.01)	0,1
**High basal sCD14 (ng/ml) levels**	25.49 (2.22 to 291.90)	0,009
**DAS28**	2.11 (0.92 to 4.84)	0,076
**Decreased ΔsCD14**		
	**Odds Ratio (95% CI)**	**p value**
**Female gender**	0.59 (0.03 to 9.58)	0,71
**Age (years)**	0.96 (0.89 to 1.03)	0,31
**Decreased ΔsCD14**	40.35 (3.02 to 537.38)	0,005
**DAS28**	2.86 (1.15 to 7.12)	0,02

Relationship between high basal sCD14 (ng/ml) serum levels or decreased ΔsCD14 and response to Methotrexate treatment in RA patients. DAS28: disease activity score estimated with 28 joint counts.

Supplementary Table 1.- Relationship between high basal sCD14 (ng/ml) serum levels or decreased ΔsCD14 and response to Methotrexate treatment in RA patients. Multivariable logistic regression analysis to estimate with a better precision the capability of sCD14 to discriminate between MTX-responder and non-responder patients. The dependent variable was response to MTX and as independent variables we included gender, age and baseline DAS28, that are well known predictors of treatment response in RA. sCD14 or ΔsCD14 were included in the model to determine their respective OR adjusted by age, gender and baseline DAS28.
